# Augmentation Mastopexy With Polyurethane-Coated Implants in the Prepectoral Plane: A 24-Month Follow-Up Study

**DOI:** 10.1093/asjof/ojag118

**Published:** 2026-06-23

**Authors:** Carmine Michele Mele, Andrea Murante, Edoardo Auriemma, Giuseppe Esposito, Luigi Castiello, Vincenzo Rullo, Santolo D’Antonio

## Abstract

**Background:**

Augmentation mastopexy aims to restore breast shape and volume in patients with ptosis. Polyurethane-coated implants may enhance implant stability in augmentation mastopexy through early tissue adhesion in the prepectoral plane; however, mid-term stability and clinical outcomes remain incompletely defined, prompting the need for the present study.

**Objectives:**

The aim of this study was to evaluate the long-term aesthetic stability, complication profile, and patient satisfaction associated with augmentation mastopexy using polyurethane-coated implants placed in the prepectoral plane, with or without selective fat grafting.

**Methods:**

From January 2019 to December 2023, 169 patients (338 breasts) underwent augmentation mastopexy with polyurethane-coated implants placed in the prepectoral plane. The surgical protocol included mastopexy, implant placement, and selective fat grafting, when needed, to optimize breast contour in patients with asymmetries or minimal soft-tissue coverage (eg, pinch test <2 cm). Patients were followed up at 1, 6, 12, 18, and 24 months postoperatively, with the primary endpoint being the nipple-to-jugular distance (NJD), used to assess breast stability. Patient satisfaction was assessed using a 5-point Likert scale at 12 months.

**Results:**

The average preoperative NJD was 27.4 cm, and postoperatively, a significant reduction was observed at 1 month (21.5 cm), with minimal increase over time (22.3 cm at 24 months), indicating stable breast positioning. A single case of capsular contracture was managed with implant replacement. Patient satisfaction was high, with 78% rating their results as “very satisfied” (Score 5).

**Conclusions:**

Augmentation mastopexy with polyurethane-coated implants placed in the prepectoral plane provides stable, long-term breast positioning and high patient satisfaction. This technique offers a reliable alternative to traditional approaches, with minimal complications and sustained aesthetic results.

**Level of Evidence: 4 (Therapeutic):**

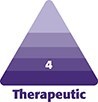

Over the years, breasts can undergo various changes, more or less significant, following pregnancies, breastfeeding, or significant weight loss.^1-3^ Breast surgery alleviates many of these conditions by addressing different grades of ptosis, asymmetries, hypoplasia, and defects of the chest wall.

However, traditional techniques often have limitations, including a high risk of ptosis recurrence, implant malposition, bottoming out, and capsular contracture. These complications can compromise the aesthetic and functional outcomes, leading to patient dissatisfaction and a need for revision surgeries.^4^

Polyurethane-coated breast implants were originally introduced in the 1970s as a solution to reduce capsular contracture through enhanced tissue integration. However, their use declined significantly in the 1980s and 1990s—particularly in the United States—because of concerns regarding late complications, such as double capsule formation, difficult explantation, and potential toxicological risks related to polyurethane degradation. In the early 2000s, a renewed interest in these implants emerged in Europe and South America, supported by improved manufacturing technologies, the use of cohesive silicone gel, and refinements in surgical technique. This “re-introduction” phase led to more favorable clinical outcomes, and polyurethane-coated implants have since been reevaluated as a reliable option in both aesthetic and reconstructive breast surgery.^5^

Although some authors have suggested that polyurethane-coated implants may reduce the incidence of capsular contracture, particularly when placed in the prepectoral plane, this remains a topic of ongoing debate. Historically, higher rates of encapsulation have been reported with prepectoral positioning compared with submuscular or dual-plane approaches, especially in the absence of proper implant coverage or surgical refinement.^6-8^ The prepectoral plane (above the pectoral muscle) has emerged as a promising alternative to submuscular implant placement. This approach has been associated with reduced postoperative pain and avoidance of muscle animation deformities. However, its role in preventing capsular contracture remains controversial. Several studies have suggested that the surface texture of the implant—particularly polyurethane—may play a more decisive role in reducing contracture risk than the implant pocket itself.^9-11^

Despite these promising individual findings, there remains a lack of comprehensive evidence on the combined benefits of polyurethane-coated implants and prepectoral placement in augmentation mastopexy.^8^ Moreover, the long-term aesthetic stability and patient satisfaction associated with this approach—which combines mastopexy and prepectoral placement of polyurethane-coated implants, with selective use of autologous fat grafting—are still not fully understood. In our protocol, fat grafting was not performed routinely but selectively, with the aim of improving breast contour symmetry or softening transitions in patients with thin tissue coverage (eg, pinch test <2 cm). The motivation for this study stems from the need to evaluate the effectiveness of this surgical strategy, particularly in addressing long-term outcomes and patient quality of life. Our goal is to provide valuable insights into the aesthetic stability, surgical outcomes, and patient satisfaction in a cohort of patients undergoing augmentation mastopexy with polyurethane-coated implants placed in the prepectoral plane. This study aims to fill a significant gap in the current literature and potentially establish a new standard of care for patients seeking both volume enhancement and correction of breast ptosis.^5,8,12^

## METHODS

From January 2019 to December 2023, a cohort of 169 patients (338 breasts) presenting with varying degrees of breast ptosis and volume hypoplasia was selected for augmentation mastopexy using round polyurethane-coated implants. This was a single-site, single-surgeon retrospective cohort study conducted by the senior author C.M.M. at a private surgical center in Naples, Italy. All procedures were performed by the same surgeon using a standardized technique. Follow-up was conducted at 1, 6, 12, 18, and 24 months postoperatively

The study included female patients aged 18 to 63 years presenting with breast ptosis, volume hypoplasia, or both, requiring augmentation mastopexy. Ptosis was classified according to the Regnault system (Grade I: mild ptosis; Grade II: moderate ptosis; and Grade III: severe ptosis).

Eligibility criteria included a stable physical and psychological health status, a BMI ≤ 30, and the absence of previous breast surgeries or conditions affecting the structural integrity of breast tissue. Only nonsmokers or individuals who ceased smoking at least 6 weeks before surgery were considered. Preoperative evaluation of the breast parenchyma was performed through physical examination, including a pinch test, and bilateral breast ultrasound in all patients to assess tissue thickness and rule out underlying lesions. Exclusion criteria encompassed a history of breast surgery, BMI > 30, active smoking, or microcirculation disorders and systemic diseases, such as diabetes or connective tissue disorders, that could impair healing or elevate surgical risk. Additionally, patients with breast pathology, including malignancies or infections, or those with unrealistic aesthetic expectations or psychological conditions potentially compromising postoperative satisfaction or adherence to follow-up protocols, were excluded (Table 1).

**Table 1.** Baseline Characteristics and Eligibility Criteria of the Study Cohort

Variable	*n* (%)/mean ± SD (range)
Total patients	37
Sex	29 females (78.4%), 8 males (21.6%)
Age (years)	31.7 ± 8.9 (18-52)
Type of deviation	I-shaped = 10 (27%) C-shaped = 15 (41%) S-shaped = 12 (32%)
Follow-up (months)	18 ± 5 (12-28)
Preoperative CT available	37 (100%)
Postoperative CT available	35 (94.6%)
Lost to follow-up	0 (0%)

CT, computed tomography; SD, standard deviation.

### Preoperative Marking

With the patient in an upright position, the midline of the chest is marked from the jugulum to the xiphoid process. A second line is traced 5 cm lateral to the jugulum, from the clavicle to the nipple–areola complex (NAC) and then extended straight down to the middle point of the inframammary fold (IMF; Point F) to define the breast meridian. The IMF is also marked, as well as the anterior axillary line. Using the Pitanguy maneuver, the point where the top of the areola will be placed is identified (Point A), and with the aid of a standard marker, the shape of the neo-areola is drawn.^13,14^ To determine the amount of skin to be excised, the Lejour maneuver is performed medially and laterally, creating the vertical limb of the reduction pattern. The length of the vertical scar is set at either 6 cm or 7 cm (Points D and E), depending on the implant projection. For implants with high projection, the vertical scar length is set at 7 cm, whereas for implants with moderate projection, it is set at 6 cm. From the caudal extreme of vertical marks, 2 horizontal lines are drawn reaching the anterior axillary line and the midline of the chest completing the Wise pattern, ensuring at least 1 cm from contralateral incisions to avoid synmastia.

To select the most appropriate round implant, the breast base is initially measured using the standard method, which involves measuring from 1 cm lateral to the chest midline to the anterior axillary line. From this measurement, the combined thickness of the medial and lateral breast tissue, determined by pinch testing, is subtracted. This calculation provides the effective implant base width.^15^

### Surgical Technique

**Figure 1.**
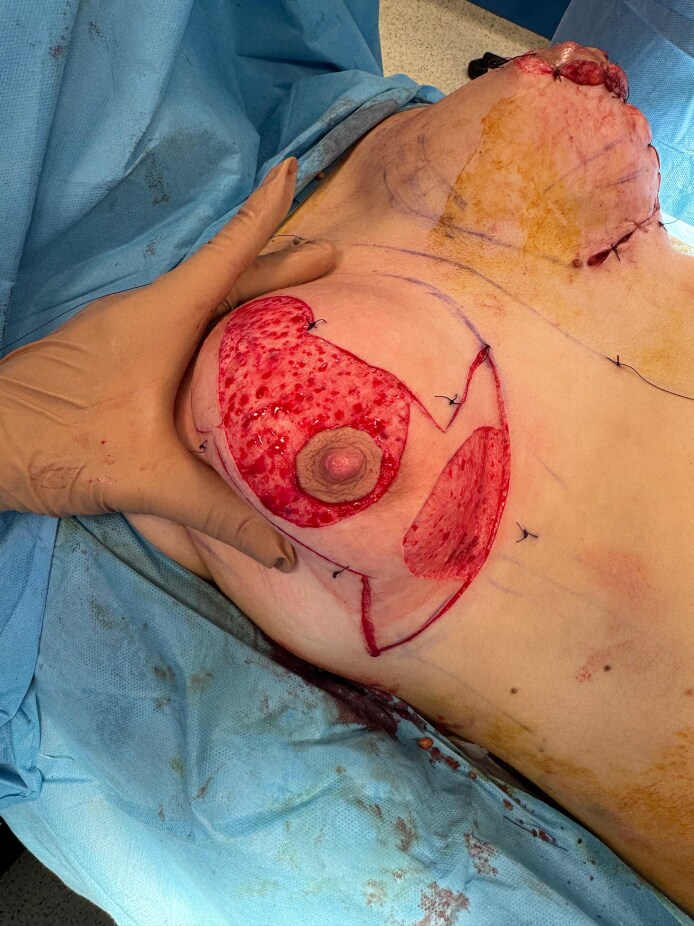
Intraoperative view in a 34-year-old woman showing the preparation of the inferior dermal flap and the prepectoral implant positioning.

The patient is positioned supine on the operating table, and after induction of general anesthesia and setting up the surgical field with antiseptic solution and sterile drapes, the neo-areola is marked on the skin using a nipple marker, and the area of the drawing is de-epithelialized, leaving the NAC in its position (Figure 1). A superomedial pedicle was employed in all cases to maintain the vascular and sensory supply to the NAC. This pedicle choice allows for safe transposition of the NAC while preserving upper pole fullness and ensuring robust perfusion, especially in cases requiring lower quadrant glandular resection.^16^

A horizontal incision is made in the de-epithelialized area 1.5 cm distal to the NAC, and an inferior dermal flap is prepared, measuring 6 cm in height, 6 cm in base, and 2 cm in thickness; this will ensure the coverage of the implant. Part of the breast tissue is removed in the lateral, medial, and retro-areolar portions, based on the amount of excess tissue, and the prepectoral pocket is prepared for implant housing as described by Eisenberg (Video).^17^ Tissue removal was mainly localized in the lower-medial and lower-lateral quadrants to optimize the implant pocket while maintaining upper pole fullness.

**Figure 2.**
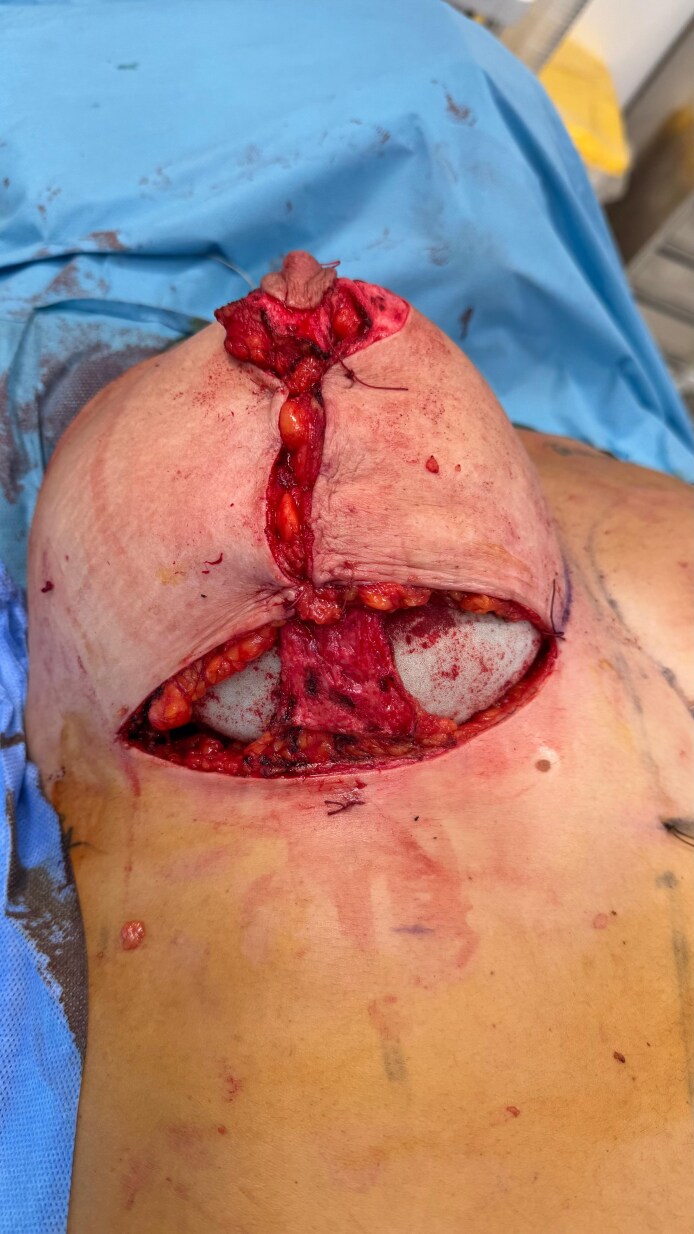
Intraoperative view in a 34-year-old woman after implant insertion, showing the polyurethane implant in place and the inferior dermal flap already anchored to the glandular tissue.

All operators changed gloves before implant placement. All patients received round polyurethane-coated silicone breast implants (Polytech Health & Aesthetics GmbH, Dieburg, Germany), placed in the prepectoral plane. The pocket was irrigated with sterile normal saline to remove any debris or residual tissue. The implant was rinsed separately with an antibiotic solution containing Gentamicin 80 mg diluted in 250 mL of normal saline before placement in the prepectoral pocket.^18,19^ The inferior dermal flap is then advanced and secured to the superior glandular plane to ensure adequate coverage of the implant (Figure 2), and the accesses are then closed with absorbable monofilament sutures (Video; Monocryl 2-0, 3-0, and 4-0; Ethicon Inc., Somerville, NJ) for the deep, superficial subcutaneous, and areolar layers, respectively.

**Figure 3.**
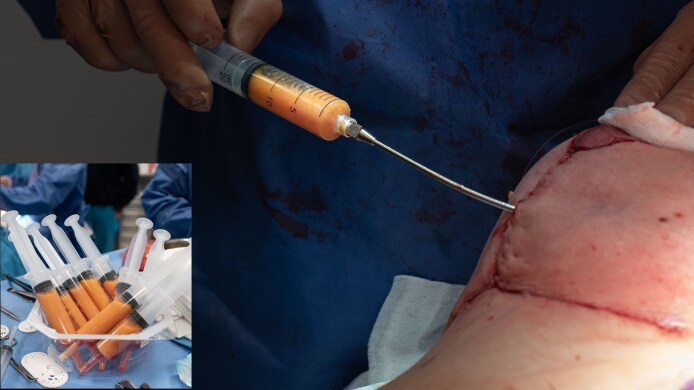
Selective lipofilling performed in a 34-year-old woman to refine breast contour and soften implant edges in patients with reduced soft-tissue thickness (eg, pinch test <2 cm).

When indicated, autologous fat grafting was selectively performed to refine breast contour and camouflage visible transitions in patients with reduced soft-tissue thickness (eg, pinch test <2 cm) or visible contour irregularities after implant placement (Figure 3).

The volume of fat injected was determined on a patient-by-patient basis, according to the degree of soft-tissue deficiency and the desired contour refinement.^20,21^ The procedure begins with the infiltration of a modified Klein solution into the medial aspect of the knee, which serves as the donor site. The solution consists of lidocaine 0.05%, epinephrine 1:1,000,000 (0.0001%), sodium bicarbonate 1%, and normal saline or lactated Ringer's solution as the diluent (98.95%), providing local anesthesia, vasoconstriction, and reduced tissue trauma. After waiting 10 min, fat is then harvested from the medial knee through liposuction and processed through decantation to isolate the adipose tissue. The purified fat is meticulously injected into the subcutaneous tissue of the breast, targeting areas where imperfections cannot be corrected solely by the implant.^22^

The primary endpoint was nipple-to-jugular distance (NJD), measured preoperatively and at each follow-up time point. Measurements were obtained using a standard digital caliper with the patient in an upright, relaxed position and arms by the sides. NJD was defined as the linear distance from the midpoint of the nipple to the jugular notch of the sternum. All measurements were performed by the operating surgeon to ensure consistency and standardization. In addition to NJD, the NAC–IMF distance was recorded as a secondary parameter to evaluate the vertical dimension of the lower breast pole, which may be influenced by implant descent or soft-tissue stretching over time. This parameter is routinely used in aesthetic breast surgery to assess the projection and stability of the lower pole.

- Preoperative measurements were obtained during the initial consultation before surgery.
- Postoperative assessments were conducted at 1, 6, 12, 18, and 24 months to monitor changes over time.

These time points were chosen based on typical recovery trajectories in breast augmentation and reconstruction, allowing for both early and long-term assessment of implant stability. The 24-month time point was particularly chosen to evaluate long-term outcomes, because it is beyond the typical period of initial postoperative healing and allows for the assessment of breast position at a stable phase.

The decision to follow patients up at these intervals was informed by the literature, as previous studies have demonstrated that breast position and implant stability can continue to change during the first 12 to 24 months postsurgery.^17^ This follow-up schedule aims to capture both immediate changes postsurgery as well as the longer-term behavior of the implant, particularly in terms of ptosis and displacement.

At each follow-up visit, the NJD was recorded, and any variations in the measurement were documented.^23^ The data were then compared with preoperative values to assess any potential descent of the breast or implant migration over time. Statistical analysis was performed using paired *t*-tests to compare the preoperative NJD with the values at 1, 6, 12, 18, and 24 months postsurgery. An analysis of variance with post hoc Tukey's test was conducted to examine differences in the NJD across different follow-up time points. Pearson's correlation was used to evaluate the relationship between changes in NJD and patient satisfaction. The results were compared with the data reported in relevant literature to determine whether prepectoral polyurethane implants offer improved long-term stability compared with smooth or textured implants.^24^ Statistical significance was defined a priori as a 2-tailed *P*-value of <.05.

As part of our follow-up protocol, patients were also asked to evaluate their level of satisfaction with the results of the procedure using a 5-point Likert scale. The scale ranged from 1 to 5, with 1 indicating “very dissatisfied,” 2 indicating “dissatisfied,” 3 indicating “neutral,” 4 indicating “satisfied,” and 5 indicating “very satisfied.” This method provided valuable insights into the subjective experience of the patients, complementing the clinical measurements of breast stability and implant position. The satisfaction ratings were recorded at 1 year postoperatively, because this time point is considered indicative of the final aesthetic outcome and recovery.

This retrospective study was conducted in accordance with the ethical standards of the institutional and/or national research committee and with the 1964 Declaration of Helsinki and its later amendments. Formal IRB approval was not required because of the retrospective and anonymized nature of the data, in accordance with local regulations. All patients provided written informed consent for the surgical procedure and for the use of anonymized clinical data and images for research and publication purposes.

## RESULTS

The average age of the patients was 40.5 years with an age range of 18 to 63. All 169 patients represented a consecutive series, and complete 24-month outcome data were available for the entire cohort. No patients were lost to follow-up. Minor variations in visit timing occurred in a few cases (±2-3 months), but full clinical data (clinical examination and standardized photographs) were obtained for all patients. No additional exclusions beyond those stated in the Methods were applied.

The implant volume ranged from 275 to 400 cc, with a mean of 345 cc (±35 cc).

The average parenchymal resection per breast was ∼45 g (range, 20-70 g), depending on the degree of ptosis and glandular excess. Among the 26 patients who received adjunctive fat grafting, on average, 45 mL of purified fat was injected per breast (range, 30-70 mL), depending on the degree of soft-tissue deficiency and desired contour refinement. Among the 169 patients treated, the following grades of ptosis, as classified by the Regnault system, were observed (Table 2):

- Grade I (mild ptosis): 36 patients (21.3%);
- Grade II (moderate ptosis): 105 patients (62.1%);
- Grade III (severe ptosis): 28 patients (16.6%).

**Table 2.** Patient Demographics and Clinical Characteristics of the Study Cohort (*n* = 169)

Parameter	Value	Notes
Age (years)	Mean 40.5 (range, 18-63)	
BMI	Mean 24.1 (range, 19.0-29.8)	All patients met inclusion criteria
Smoking status	Nonsmokers or ceased ≥6 weeks preoperative	Per protocol
Follow-up duration	24 months	100% completion
Implant volume (cc)	Mean 345 (range, 275-400)	Round polyurethane implants
Parenchymal resection (g)	Mean 45 (range, 20-70)	Per breast
Adjunctive fat grafting	26 patients (15.4%)	Selective, not routine
Ptosis Grade I	36 patients (21.3%)	Mild
Ptosis Grade II	105 patients (62.1%)	Moderate
Ptosis Grade III	28 patients (16.6%)	Severe
Revisions included?	No	Only primary cases

Among the 169 patients, we recorded a single case of capsular contracture (Baker grade III) at 14 months postoperatively that required implant replacement. Additionally, 1 case (0.6%) of postoperative breast implant infection was recorded, which required surgical revision and implant replacement.

Furthermore, 2 cases (1.2%) of superficial wound dehiscence at the vertical scar occurred within the first 2 weeks postoperatively. These were managed with local wound care and healed without further surgical intervention. Minor areolar delayed healing was observed in 3 patients (1.8%), all of which resolved conservatively. No cases of hematoma, seroma, or implant malposition were reported. Among the 26 patients who received adjunctive fat grafting, no major complications were observed. Specifically, there were no cases of palpable oil cysts, clinically evident steatonecrosis, or infections at the donor or recipient sites. Minor volume resorption was noted in some cases during the first 6 months, but no patients required revision fat grafting.

Among the 169 patients included in the study, the preoperative mean NJD was 27.4 cm (± 2.3 cm), reflecting the presence of breast ptosis.

The mean preoperative NJD was 27.4 ± 2.3 cm. Postoperatively, NJD decreased to 21.5 ± 1.9 cm at 1 month and showed only minimal changes over time, measuring 21.8 ± 1.8 cm at 6 months, 22.1 ± 1.7 cm at 12 months, 22.3 ± 1.6 cm at 18 months, and 22.4 ± 1.5 cm at 24 months.

The reduction in NJD was statistically significant at all postoperative time points compared with preoperative value: 1 month: *P* < .001; 6 months: *P* < .001; 12 months: *P* < .001; 18 months: *P* < .001; and 24 months: *P* < .001.

Although the NJD increased slightly from 21.5 cm at 1 month to 22.3 cm at 18 months, it remained significantly reduced compared with preoperative measurements at all time points (Table 3).

**Table 3.** Nipple-to-Jugular Distance (NJD) Measured Preoperatively and at All Postoperative Time Points Through 24 Months.

Follow-up period	Mean NJD (cm)	Standard Deviation (cm)	Statistical Significance
Preoperative	27.4	±2.3	—
1 month	21.5	±1.9	*P* < .0001
6 months	21.8	±1.8	*P* < .0001
12 months	22.1	±1.7	*P* < .0001
18 months	22.3	±1.6	*P* < .0001
24 months	22.4	±1.5	*P* < .0001

*P*-values are calculated in comparison with preoperative values.

To specifically evaluate postoperative stability, NJD measurements at 6, 12, 18, and 24 months were compared with the 1-month postoperative value. No statistically significant differences were observed at any subsequent follow-up time point, indicating stable breast position over time (Table 4).

**Table 4.** Pairwise Comparisons of Nipple-to-Jugular Distance (NJD) and Nipple-to-Inframammary Fold (NAC–IMF) Measurements at 6, 12, 18, and 24 Months vs the 1-month Postoperative Value, Performed to Assess Postoperative Stability

Time point	NJD (mean ± SD)	*P* vs 1 month (NJD)	NAC–IMF (mean ± SD)	*P* vs 1 month (NAC–IMF)
1 month	21.5 ± 1.9	—	6.6 ± 0.4	—
6 months	21.8 ± 1.8	*P* = .28	6.7 ± 0.4	*P* = .41
12 months	22.1 ± 1.7	*P* = .14	6.8 ± 0.4	*P* = .22
18 months	22.3 ± 1.6	*P* = .08	6.8 ± 0.5	*P* = .19
24 months	22.4 ± 1.5	*P* = .06	6.9 ± 0.5	*P* = .03*

*P*-values are based on pairwise comparisons vs the 1-month postoperative measurement using the statistical method described in the Methods section. SD, standard deviation.

^*^Small absolute change (0.2 cm), clinically negligible.

In addition to NJD, the NAC–IMF distance was recorded to assess lower-pole stability. The mean preoperative NAC–IMF distance was 6.7 ± 0.5 cm. Postoperatively, this measurement remained stable, measuring 6.6 ± 0.4 cm at 1 month, 6.7 ± 0.4 cm at 6 months, 6.8 ± 0.4 cm at 12 months, 6.8 ± 0.5 cm at 18 months, and 6.9 ± 0.5 cm at 24 months.

The changes in NAC–IMF distance were small and mostly were not statistically significant when compared with the preoperative value: 1 month: *P* = .12; 6 months: *P* = .85; 12 months: *P* = .09; 18 months: *P* = .17.

A minimal difference reached statistical significance at 24 months (*P* = .04), although the magnitude of change (0.2 cm) remained clinically negligible. Overall, the NAC–IMF variations confirmed the stability of the lower pole and implant position over time (Table 5).

**Table 5.** Nipple-to-Inframammary Fold (NAC–IMF) Distance Measured Preoperatively and at Postoperative Follow-Up Time Points Through 24 Months

Time point	NAC–IMF distance (cm ± SD)	*P*-value
Preoperative	6.7 ± 0.5	—
1 month	6.6 ± 0.4	.12
6 months	6.7 ± 0.4	.85
12 months	6.8 ± 0.4	.09
18 months	6.8 ± 0.5	.17
24 months	6.9 ± 0.5	.04

*P*-values are calculated in comparison with preoperative values. SD, standard deviation.

Pairwise comparison of NAC–IMF measurements at 6, 12, 18, and 24 months vs the 1-month postoperative value showed minimal changes over time. A small difference reached statistical significance at 24 months (*P* = .04); however, the magnitude of variation remained clinically negligible, supporting lower-pole stability (Table 4).

**Figure 4.**
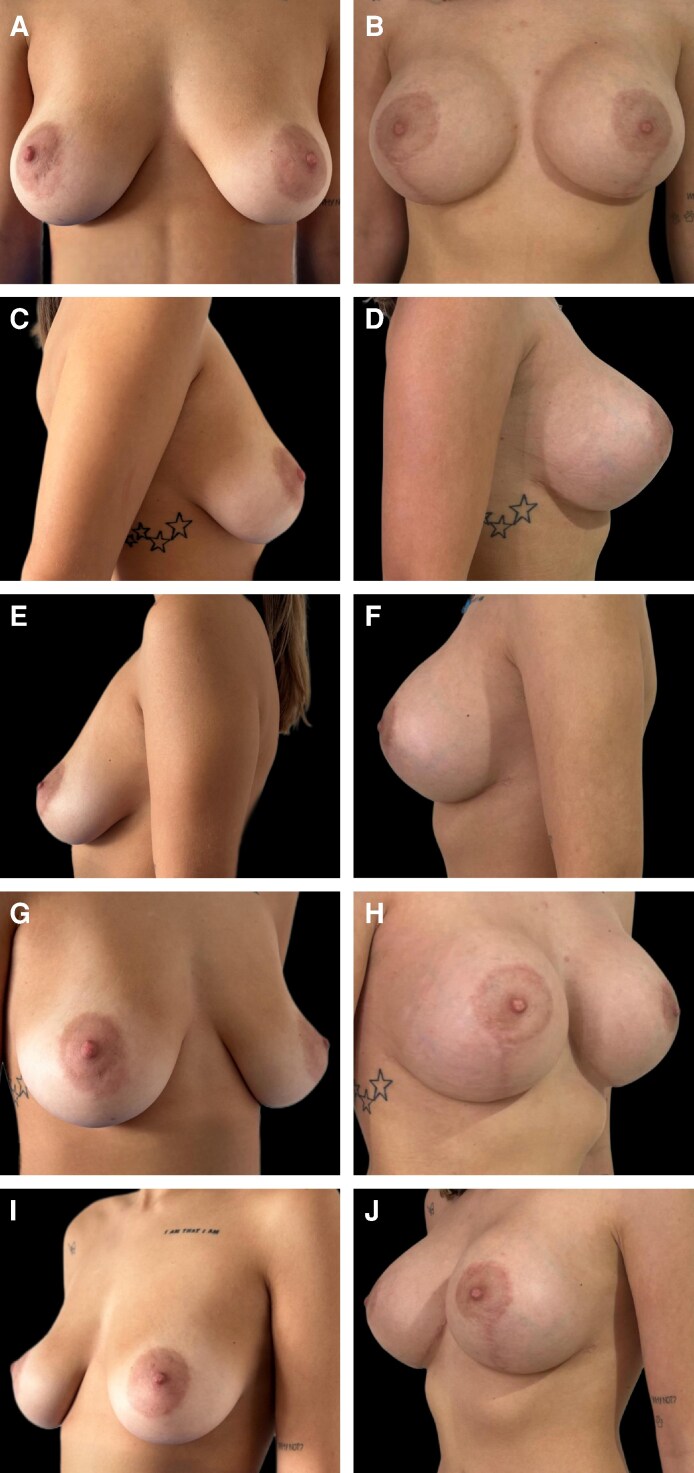
(A, C, E, G, I) Preoperative views of a 24-year-old woman presenting with moderate bilateral breast ptosis (Grade II) and glandular asymmetry. (B, D, F, H, J) Postoperative views at 24-month follow-up after prepectoral augmentation-mastopexy using 325 cc polyurethane implants and a superomedial pedicle. The upper pole contour remained full, with preserved NAC–IMF distance and high patient satisfaction.

**Figure 5.**
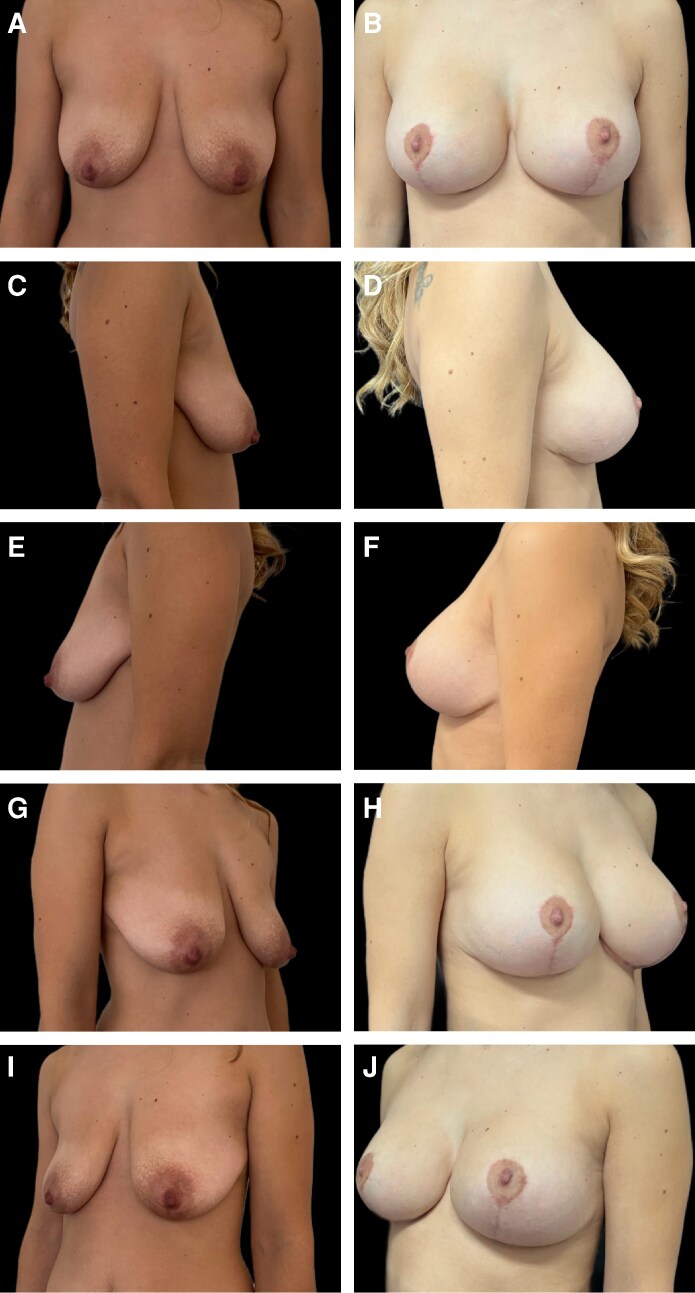
(A, C, E, G, I) Preoperative views of a 38-year-old patient presenting with severe glandular descent, Grade III breast ptosis, and marked asymmetry. (B, D, F, H, J) Postoperative views at 24-month follow-up after mastopexy with 375 cc polyurethane implants. The results demonstrate improved breast projection, centralized nipple–areola complex position, and a natural, harmonious breast shape.

Patient-reported satisfaction at 12 months is summarized in Table 6. The mean satisfaction score was 4.6. Most patients reported high satisfaction, with 78% rating their outcome as very satisfied and 15% as satisfied. A small proportion of patients reported neutral (4%) or dissatisfied outcomes (Scores 1 and 2: 3%). Representative clinical outcomes are illustrated in Figures 4 and 5.

**Table 6.** Patient-Reported Satisfaction Scores (5-Point Likert Scale) at 12 Months

Satisfaction score	No. of patients (*n* = 169)	Percentage of total
5 (very satisfied)	132	78
4 (satisfied)	26	15
3 (neutral)	7	4
2 (dissatisfied)	3	2
1 (very dissatisfied)	1	1
Mean score	4.6	—

## DISCUSSION

In this study, we evaluated the outcomes of augmentation mastopexy with polyurethane-coated implants in the prepectoral plane and found stable breast position, minimal lower-pole descent, high patient satisfaction, and a low complication rate at 24 months.

### Advantages of Polyurethane Implants and Prepectoral Plane Approach

The prepectoral plane avoids pectoralis muscle dissection, reducing postoperative discomfort and eliminating animation deformity. These features, along with the bioadhesive behavior of polyurethane implants, contribute to predictable implant stability and a faster recovery compared with traditional submuscular techniques.^25^ Although some surgeons advocate routine drain placement with polyurethane implants, recent evidence suggests that a meticulous pocket dissection and careful hemostasis allow safe drainless surgery.^26-29^ Our intent is not to dismiss submuscular advancements but to present an alternative that leverages different biomechanical principles. Modern submuscular “rapid recovery” techniques minimize muscle trauma, yet they cannot eliminate animation deformity. Prepectoral placement avoids this issue entirely while maintaining implant stability, particularly when supported by polyurethane foam. Although the subfascial plane is also a valid option for polyurethane implant placement, in this series, we intentionally adopted a purely prepectoral approach. Polyurethane-coated implants rely on rapid bioadhesion to the surrounding tissues, and the prepectoral plane provides a broad, stable surface that facilitates this process. In our experience, the additional fascial elevation required for a subfascial pocket does not confer superior stability and may prolong operative time without improving clinical outcomes. In patients with limited soft-tissue thickness (pinch test <2 cm), combining a dermal flap with the biointegrative behavior of polyurethane implants provided reliable coverage, minimizing implant visibility or palpability. This approach allows preservation of muscle function and avoids animation deformity, which remains a limitation of the submuscular pocket, particularly in thin patients. Nevertheless, several studies have shown that in the short and mid-term, polyurethane implants may offer lower capsular contracture rates compared with traditional textured implants.^8,12,30^

It is important to note that over time, the polyurethane foam coating undergoes degradation, and some evidence suggests that its long-term performance may approximate that of other surface types after the polyurethane has been absorbed.^30^

Histological studies confirm that polyurethane foam undergoes gradual hydrolytic degradation over 6 to 12 months, with byproducts metabolized through macrophage activity. Despite this process, no clinical evidence currently supports systemic accumulation or increased late complications.^31^

Moreover, although the risk of breast implant–associated anaplastic large cell lymphoma (BIA-ALCL) remains low, highly textured surfaces—including polyurethane—have been associated with an increased relative risk when compared with smooth implants.

Recent epidemiological studies have further clarified the association between highly textured implant surfaces and BIA-ALCL. In the Australian and New Zealand epidemiological study by Magnusson et al, the highest relative risk was observed for Grade 4 surface breast implants.^32^

These factors underline the importance of long-term follow-up and balanced evaluation of benefits vs risks when selecting polyurethane implants for aesthetic or reconstructive procedures.

Polyurethane-coated implants have long been recognized for their ability to achieve superior biointegration with surrounding tissues, reducing the risk of capsular contracture and implant displacement compared with traditional smooth or textured implants. The prepectoral plane placement of the implant further minimizes the need for pectoralis major muscle dissection, which has been associated with less postoperative pain and a reduced risk of complications such as seromas and hematomas.^33,34^ The prepectoral approach avoids pectoralis muscle dissection and eliminates animation deformity.^35^

The primary endpoint of this study, the NJD, was used as a reliable marker for breast ptosis and implant position. Preoperative measurements showed a mean NJD of 27.4 cm (±2.3 cm), indicating moderate-to-severe ptosis in all patients. Postoperatively, there was an immediate and statistically significant reduction in this distance at 1 month (21.5 cm ± 1.9 cm), followed by a slight increase during the follow-up period, from 21.5 cm at 1 month to 22.3 cm at 18 months. However, the difference between preoperative and postoperative measurements remained statistically significant at all follow-up points (*P* < .05), indicating stable and lasting improvements in breast position.

The progressive but minimal increase in NJD over 24 months reflects natural tissue settling without clinically relevant ptosis recurrence.

However, it is important to note that meaningful comparisons with the existing literature are not possible. The studies we reviewed did not provide directly comparable data, because they focused on different surgical techniques, implant types, or patient cohorts. As a result, any potential comparisons are limited, and we could not identify studies with overlapping methodologies or patient characteristics that would allow for a valid, direct comparison.

Therefore, although our findings suggest improved long-term stability in breast position with polyurethane-coated implants, further research with comparable cohorts is needed to substantiate these results. These findings align with previous studies demonstrating that the prepectoral approach to implant placement can achieve stable and aesthetically favorable outcomes in breast augmentation and mastopexy procedures.^8^ The selective use of autologous fat grafting in our protocol was intended to optimize aesthetic outcomes without adding unnecessary steps to all cases. In patients with minor asymmetries, visible transition zones, or reduced soft-tissue thickness (eg, pinch test <2 cm), lipofilling allowed for improved implant camouflage and a more harmonious breast contour. This approach contributes to enhanced aesthetic satisfaction while maintaining surgical efficiency.

The high satisfaction rate (mean score 4.6/5) reflects both the stability of the breast footprint and the natural contour achieved with selective lipofilling.

These results suggest that the combination of polyurethane-coated implants, the prepectoral approach, and immediate fat grafting contributes not only to stable clinical outcomes but also to high levels of patient satisfaction. The vast majority of patients reported moderate-to-high satisfaction, reinforcing the overall success of the technique.

Despite the positive outcomes, it is important to note that 1 case of capsular contracture was observed, which was successfully managed with implant replacement surgery. The occurrence of capsular contracture in this case highlights the need for continued monitoring, even with the use of polyurethane-coated implants. However, the overall low incidence of complications in this study supports the safety and effectiveness of the described technique.

Recent literature has further investigated the long-term behavior and safety profile of polyurethane-coated implants. Additionally, in a previous study, the same authors highlighted the importance of patient selection and surgical precision in mitigating the risks associated with highly textured surfaces, including BIA-ALCL.^30^ These findings underscore the need for continued surveillance and technical optimization when using polyurethane-coated devices, even in light of their favorable short- and medium-term outcomes.

An extended follow-up beyond 24 months is currently underway: our earliest cases, operated in 2019, now exceed 48 months with no late capsular contracture, bottoming out, or other major complications observed to date. These long-term data will be presented in a separate analysis once the sample size becomes sufficiently robust.

Despite the promising results, our study has several limitations that should be considered when interpreting the findings. The primary limitation is the relatively small sample size (169 patients), which may not be sufficiently representative to generalize the results to a larger population. In the future, a multicenter study with a larger cohort would be beneficial to strengthen the external validity of our results.

Another limitation is the potential measurement bias, as all anthropometric measurements (NJD and NAC–IMF) were performed by the operating surgeon. Although this ensured consistency across all time points, it may introduce observer-related bias and should be considered when interpreting the findings.

Additionally, the 24-month follow-up, although adequate for assessing medium-term outcomes, may not be long enough to evaluate late complications, such as capsular contracture or further changes in nipple position. A longer follow-up period (beyond 2-3 years) would be crucial to assess the long-term stability of this technique.

Revision surgeries were excluded from this series in order to maintain a homogeneous sample and avoid confounding factors related to previous surgical scarring, altered tissue planes, or capsular fibrosis. Although we agree that secondary cases are valuable in assessing the robustness of any technique, we intentionally focused on primary cases only to objectively evaluate the intrinsic stability and complication profile of the prepectoral polyurethane approach without the bias of previous surgical alterations. Future studies will explore the application of this technique in revision settings.

Another limitation is the absence of a control group with an alternative technique. Although comparisons with literature data suggest that our results are superior to those of other prepectoral augmentation mastopexy techniques using smooth or microtextured implants, the lack of a randomized control group limits the strength of our conclusions.

Finally, although the patient satisfaction assessment using a Likert scale provides useful insight, it may not fully capture all the psychological and physical aspects of the postoperative experience. More complex tools, such as validated quality of life questionnaires or psychological assessments, could provide more detailed information.

### Comparative Context With Traditional Techniques

Although our series lacks a built-in control group, several large published cohorts provide reference values for augmentation mastopexy performed with smooth or microtextured implants in submuscular or dual-plane pockets.

- In the meta-analysis by Khavanin et al (23 studies, 1826 breasts), overall complications reached 13.1%, with recurrent ptosis 5.2% and capsular contracture 3.0% at a mean 18-month follow-up.^36^
- Bresnick reported capsular contracture of 1.6% to 5.4% at 12 months in 322 dual-plane cases with smooth implants, depending on incision site.^37^
- Swanson documented a 15.1% overall revision/complication rate in 47 vertical augmentation mastopexies with smooth implants, despite 91.5% of patients reporting their expectations as met.^38^

In comparison, our polyurethane, prepectoral technique produced a 0.6% capsular-contracture rate, no bottoming out or implant malposition, and a total complication rate of 3.6% at 24 months. Although the comparison is not randomized, these figures suggest that combining a biointegrative polyurethane surface with a prepectoral pocket, dermal support and selective lipofilling may provide greater lower-pole stability and fewer unplanned re-interventions than the techniques most commonly reported in the literature.

## CONCLUSIONS

Augmentation mastopexy with polyurethane-coated implants in the prepectoral plane demonstrated stable breast positioning, low complication rates, and high patient satisfaction at 24 months of follow-up. This technique, combined with selective autologous fat grafting, appears to offer a safe and effective alternative to traditional approaches. Further studies with larger cohorts and extended follow-up are needed to confirm these findings.
